# fMRI correlates for low frequency local field potentials appear as a spatiotemporal dynamic under multiple anesthetic conditions

**DOI:** 10.1186/1471-2202-13-S1-O19

**Published:** 2012-07-16

**Authors:** Garth J Thompson, Wen-Ju Pan, Matthew E Magnuson, Shella D Keilholz

**Affiliations:** 1 Biomedical Engineering, Emory University and Georgia Institute of Technology, Atlanta, GA 30306, USA

## 

**Figure 1:**
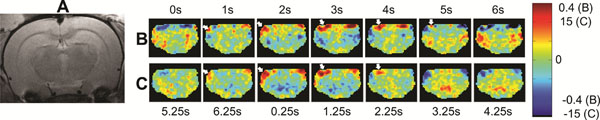
**A.** A coronal image of a rat’s brain in the same plane as the fMRI images used in this study. **B.** (med) *r*_t_ between LFP and fMRI at each voxel, times listed are the time shift of LFP prior to fMRI. **C.** (med) fMRI pattern from Majeed et al. algorithm [5], times listed are arbitrary, so they are shifted to match (B).

In the previous decade, interest in the “functional connectivity” of the brain has greatly increased, but the nature of the signal underlying derived predictive metrics remains poorly understood [1]. A typical study uses functional magnetic resonance imaging (fMRI) and calculates regions of correlated low-frequency activity or “functional networks” when no task is being performed, the “resting state”. However, unlike traditional block/event based fMRI, the spontaneous fluctuations that determine such networks may not relate to a standard “hemodynamic response” to neural activity [2] and may be task and brain region dependent [1]. Ten rats were anesthetized with either isoflurane (iso) or dexmedetomidine (med). Each rat had simultaneous local field potentials (LFP) [3] recorded from implanted electrodes in bilateral primary somatosensory cortex (SI) simultaneously with single-slice fMRI of SI [4]. After preprocessing, signals were filtered to regions of significant spectral coherence (0.04-0.18Hz iso, 0.05-0.3Hz med). Pearson correlation (*r*_t_) was calculated between LFP signals at time shifts -10s to 10s relative to fMRI, at every fMRI voxel (Figure 1B). Instead of a simple hemodynamic response, the LFP correlates appeared both to have a component of spatial propagation (Figure 1B, white arrows), and alternation between positive and negative correlation. This was observed using both anesthesias and suggests that LFPs in coherent frequencies do not simply reflect local activation, but may instead be part of a large scale dynamic process. Using an fMRI-based algorithm validated in both anesthetized rats and awake humans [5], a spatiotemporal dynamic was produced that was highly similar to *r*_t_ (Figure 1C). Spatial correlation (*r*_s_) between the two types of pattern reached a maximum at approximately the same shift between patterns in all rats, mean *r*_s_ = 0.25 (med) and mean *r*_s_ = 0.23 (iso), with mean *r*_s_ > 0.10 indicating significance at p < 0.05 when using boot-strapping and correcting for multiple comparisons [6]. These results suggest that the neural basis of functional networks may be more complex than a simple hemodynamic response and possibly contains contributions from large-scale neuromodulatory processes.
